# The developmental trajectories of spatial skills in middle childhood

**DOI:** 10.1111/bjdp.12380

**Published:** 2021-05-18

**Authors:** Alex Hodgkiss, Katie A. Gilligan‐Lee, Michael S.C. Thomas, Andrew K. Tolmie, Emily K. Farran

**Affiliations:** ^1^ Department of Education University of Oxford UK; ^2^ School of Psychology University of Surrey Guildford UK; ^3^ Department of Psychological Sciences Birkbeck, University of London UK; ^4^ Department of Psychology and Human Development UCL Institute of Education, UCL London UK; ^5^ School of Psychology University of Surrey Guildford UK

**Keywords:** children, development, extrinsic, intrinsic, spatial skills

## Abstract

The multidimensional structure of spatial ability remains a debated issue. However, the developmental trajectories of spatial skills have yet to be investigated as a source of evidence within this debate. We tested the intrinsic versus extrinsic and static versus dynamic dimensions of the Uttal et al. (2013, *Psychol. Bull*., *139*, 352) typology in relation to spatial development. Participants (*N* = 184) aged 6–11 completed spatial tasks chosen to measure these spatial dimensions. The results indicated that the developmental trajectories of intrinsic versus extrinsic skills differed significantly. Intrinsic skills improved more between 6 and 8 years, and 7 and 8 years, than extrinsic skills. Extrinsic skills increased more between 8 and 10 years than intrinsic skills. The trajectories of static versus dynamic skills did not differ significantly. The findings support the intrinsic versus extrinsic, but not the static versus dynamic dimension, of the Uttal et al. (2013, *Psychol. Bull*., *139*, 352) typology.

**Statement of contribution**

***What is already known on this subject?***

- The dimensional structure of spatial ability is a debated issue.
- The Uttal et al. (2013) model proposes that spatial thinking is comprised of two dimensions.
- There is a scarcity of developmental findings assessing the validity of these dimensions.

***What does this study add?***

- The developmental trajectories of intrinsic versus extrinsic skills differ significantly.
- The developmental trajectories of static versus dynamic skills do not differ significantly.
- Spatial skill developmental trajectories support the intrinsic versus extrinsic dimension only.

## Background

Spatial cognition is the processing, representation, comparison, and transformation of spatial information. Spatial cognition was first distinguished from general intelligence in the 1930s, and since this time, attempts at defining a typology for spatial thinking have led to the emergence of many contrasting typologies (for example, Linn & Petersen, 1985). While these models all assume that spatial cognition is composed of several dimensions, the precise nature of these remains hotly debated. To date, evidence for different spatial dimensions has been mostly derived from psychometric analyses, as well as findings from experimental cognitive psychology and cognitive neuroscience. However, the similarities and differences between spatial skill developmental trajectories in childhood are currently an untapped source of evidence for separable spatial dimensions. The aim of the current study was therefore to investigate the developmental trajectories of different spatial dimensions through middle childhood, as a means of testing the proposed dimensions of a prominent spatial typology.

### Typology of spatial thinking

**Figure 1:**
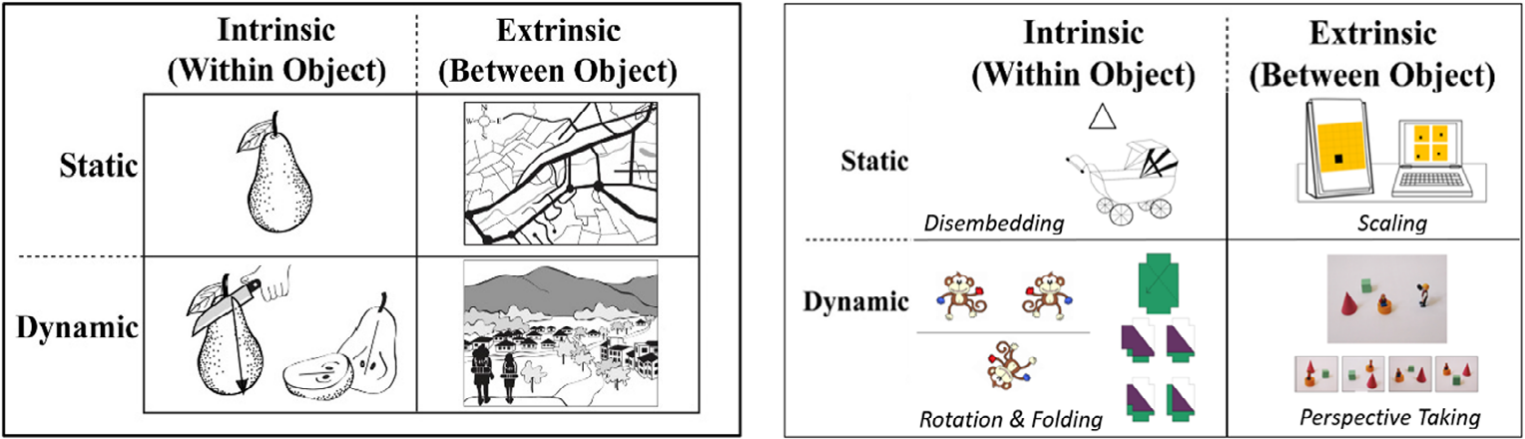
On the left, the Uttal et al. (2013) framework of spatial thinking (source: Newcombe, 2018). On the right, the tasks included in this study to assess each of the Uttal et al. (2013) spatial dimensions.

Among the spatial typologies proposed, one theory‐driven approach has gained significant support. Uttal et al. (2013), and Newcombe and Shipley (2015), proposed a classification of spatial thinking which distinguishes skills as being intrinsic versus extrinsic along one dimension, and static versus dynamic, along the other (see Figure 1). Intrinsic skills are within‐object, that is, pertaining to the size and orientation of an object, its parts, and their relationships, and extrinsic skills are between‐object, that is, relating to the relationship between objects, and between objects and their frames of reference. Dynamic skills involve movement or transformation, for example, imagined rotation or folding, whereas static skills do not, and involve object representation only.

Previous evidence supports the intrinsic versus extrinsic dimension. From an evolutionary perspective, it has been proposed that humans have two primary spatial functions, tool use, and navigation, each with a distinct evolutionary and neural basis (Newcombe, 2018). Tool use involves intrinsic spatial relations, whereas navigation involves the extrinsic coding of relations between objects (e.g., the car is next to the tree) and between objects and wider frames of reference (e.g., the car is on the west side of the mountain). Behavioural evidence also supports the intrinsic–extrinsic distinction in adults. Hegarty and Waller (2004) performed a confirmatory factor analysis (CFA) of three intrinsic tasks (mental rotation) and three extrinsic tasks (perspective taking measures requiring visualisation of a scene from different vantage points). It was revealed that a two‐factor model, that is, intrinsic versus extrinsic, fitted the data better than a one‐factor model, that is, all tasks measuring a single spatial skill (Hegarty & Waller, 2004). There is also evidence that real‐world navigation is more closely related to spatial perspective taking than mental rotation (Kozhevnikov, Motes, Rasch, & Blajenkova, 2006), as is self‐reported sense of direction (Kozhevnikov & Hegarty, 2001).

In child populations, Mix, Hambrick, Satyam, Burgoyne, and Levine (2018) tested Uttal et al.'s (2013) classification of spatial skills using CFA in 6‐, 9‐, and 12‐year‐olds. At 6 and 9 years, a two‐factor model distinguishing between intrinsic skills and extrinsic skills fitted the data better than a one‐factor model. At 12 years, none of the more complex models fitted better than a one‐factor model. However, a one‐factor model did not fit the data well, suggesting that spatial cognition does not have a unitary structure for this age group. The lack of support for the more complex models (i.e., intrinsic vs. extrinsic) for the older children in Mix et al. (2018) may relate to the particular choice of spatial tasks for this age group within this study. In line with this, using different spatial tasks than Mix et al. (2018), Vander Heyden, Huizinga, Kan, and Jolles (2016), and Heil (2018) showed through CFA that a two‐factor model distinguishing between intrinsic (mental rotation, mental folding) and extrinsic (navigating through a route after a change of perspective) skills fitted the data better than a one‐factor model, for children aged 10.5 years.

In terms of brain‐based evidence, data from adults suggest that intrinsic and extrinsic tasks are associated with activation of dissociable but overlapping neural systems. Intrinsic tasks (e.g., mental rotation) activate the right temporo‐parietal cortices and visuospatial cortical areas, whereas extrinsic tasks (e.g., perspective taking) activate the left temporo‐parietal cortices and motor areas (Wraga, Shephard, Church, Inati, & Kosslyn, 2005; Zacks, Vettel, & Michelon, 2003). FMRI research indicates that perspective taking shows similar patterns of brain activation to navigation, such that both activate the retrosplenial cortex and hippocampus (Lambrey, Doeller, Berthoz, & Burgess, 2012).

There is less convincing evidence for the static versus dynamic dimension. Assessing the static versus dynamic dimension is confounded by the fact that, for many tasks, static skills may be a necessary prerequisite to dynamic skills; that is, it is necessary to encode a shape before mentally transforming it. In adults, evidence for the static versus dynamic distinction comes from behavioural studies. For example, adults can be separated into object visualizers, who have significantly higher performance on static tasks than dynamic tasks, and spatial visualizers, who show the opposite pattern (Kozhevnikov, Kosslyn, & Shephard, 2005). However, in child‐based studies, the aforementioned CFA study by Mix et al. (2018) found no support for a two‐factor static‐dynamic model. No other known studies explore the static versus dynamic dimension in childhood.

To summarise, there is convincing evidence of an intrinsic versus extrinsic distinction in spatial thinking. For static versus dynamic skills, there is little evidence that these dimensions psychometrically dissociate. However, if static skills (perceiving and encoding static images) are a prerequisite for dynamic skills, static skills may show earlier development and an earlier plateau, than dynamic skills. We hypothesize that the current study may therefore reveal age‐based differences along the static and dynamic dimension of spatial thinking, which has not been previously evident within psychometric analyses. In the current study, spatial tasks were selected based on Uttal et al.'s (2013) theoretical framework of spatial cognition (see also Newcombe & Shipley, 2015).

### The development of spatial skills

Whilst the developmental structure of the Uttal et al. (2013) model has yet to be directly tested, below we provide a short summary of the current literature on the development of individual spatial skills. Given that there is currently a larger evidence base for the intrinsic versus extrinsic dimension, we have organized the literature along these lines.

#### Intrinsic skills

The most simplistic form of intrinsic spatial representation requires the ‘coding of spatial features of objects, including their size and the arrangement of their parts’ (Newcombe & Shipley, 2015, p. 6), that is, encoding intrinsic‐static representations. The Children’s Embedded Figures Task (CEFT) measures intrinsic‐static spatial thinking and requires individuals to identify a shape that is embedded in a more complicated image (Ekstrom, French, & Harman, 1976; Okamoto, Kotsopoulos, McGarvey, & Hallowell, 2015; Witkin, Otman, Raskin, & Karp, 1971). There is evidence that children successfully complete preschool versions of the CEFT by 3 years, and performance continues to improve on the main version of the CEFT between 3 and 5 years and also until 10 years (Busch, Watson, Brinkley, Howard, & Nelson, 1993; Witkin et al., 1971).

Other forms of intrinsic spatial thinking require visualizing and mentally transforming 2D and 3D objects (Newcombe & Shipley, 2015), that is, intrinsic‐dynamic skills. These tasks can be further subdivided into rigid and non‐rigid transformations. For rigid transformations, distances between points on an object are preserved during the transformation, for example, mental rotation. Data from violation of expectation paradigms indicate that early precursors of successful mental rotation emerge at 16 months (Frick & Wang, 2014). Results from studies using imagined rotations, only report above chance accuracy on mental rotation tasks from 5 years of age (Broadbent, Farran, & Tolmie, 2014; Frick, Hansen, & Newcombe, 2013; Marmor, 1975, 1977); Okamoto‐Barth & Call, 2008). Crescentini, Fabbro, and Urgesi (2014) found that basic 2D rotation skills improve significantly between 7 and 8 years, with no significant improvement thereafter. However, for 3D rotation, performance continues to improve until 10 (Vander Heyden et al., 2016) or even 13 years (Johnson & Meade, 1987). Similar findings have been reported for other rigid transformation tasks, for example, Child Mental Transformation Task (Levine, Huttenlocher, Taylor, & Langrock, 1999). There is less research exploring the development of non‐rigid transformations where the distance between points changes as the transformation occurs (Atit, Shipley, & Tikoff, 2013). There is evidence that by 5 years the majority of children demonstrate above chance performance on mental folding (imagining an object after it has been folded) which improves until 7–8^1^ years (Harris, Hirsh‐Pasek, & Newcombe, 2013).

Overall, the findings for the development of intrinsic‐dynamic skills indicate that the precursors of successful intrinsic‐dynamic spatial thinking are evident in infancy; 2D intrinsic‐dynamic skills, measured using both rigid and non‐rigid transformations, continue to develop until at least 7–8 years; and 3D rigid intrinsic‐dynamic skills continue to develop through later childhood. In the current study, two intrinsic‐dynamic tasks were included to reflect rigid and non‐rigid transformations.

#### Extrinsic skills

Considering next extrinsic skills, extrinsic‐static spatial thinking involves the coding of object locations in relation to other objects, spatial frameworks, or landmarks. Historically, these skills were assessed through horizontal and vertical invariance tasks. For example, performance on the Rod and Frame Test, which tests the ability to accurately code horizontal and vertical dimensions of a rod as defined by gravity, while ignoring the reference of a tilted frame, improves with age from 4 years until adulthood (Bagust, Docherty, Haynes, Telford, & Isableu, 2013; Haywood, Teeple, Givens, & Patterson, 1977; Newcombe & Shipley, 2015). Spatial scaling tasks, which measure the ability to successfully map encoded distances between different sized spaces, also assess extrinsic‐static spatial skills. In localisation paradigms, participants are shown the location of a target and are asked to find the corresponding location on a scaled target space. Using a 2D localization task, Frick and Newcombe (2012) reported that children’s scaling ability improves with age from 3 to 6 years. In more naturalistic environments, Vasilyeva and Huttenlocher (2004) found that 90% of 5‐year‐olds but only 60% of 4‐year‐olds could successfully place objects on a rectangular rug using a 2D map. The spatial scaling data presented in the current study are based on a previously published discrimination paradigm (Gilligan, Hodgkiss, Thomas, & Farran, 2018) where participants determined which one of four referent maps corresponded to a scaled version of a model map. It was found that scaling performance improved between 5 and 8 years, with no significant improvement thereafter.

A second component of extrinsic spatial thinking involves visualizing an environment from a different position (Uttal et al., 2013), that is, extrinsic‐dynamic spatial skills. Perspective taking tasks require the ability to use an object‐based (allocentric) reference frame, to represent a viewpoint that differs from one’s own (Frick, Möhring, & Newcombe, 2014). Perspective taking is proposed to develop in two stages. During Level 1, a child understands that another person can see something different to themselves, but cannot imagine exactly what can be seen from a contrasting view point (Flavell, Everett, Croft, & Flavell, 1981; Newcombe & Huttenlocher, 2003). Level 1 skills have been reported in children from 24 months (Moll & Tomasello, 2007). Level 2 perspective taking is the ability to determine *exactly how* another person would perceive an object, or array of objects, from a different perspective. Level 2 perspective taking emerges from around 4–5 years and continues to develop until at least 8 years (Frick et al., 2014; Pillow & Flavell, 1986).

The literature reviewed above suggests that there may be subtle differences in the early developmental profiles of each spatial dimension. However, few studies compare performance on different measures of spatial thinking with the same sample of participants and there is limited evidence on the development of spatial skills beyond 8 years.

### Gender differences in spatial task performance

Previous studies of gender differences in spatial skill in middle childhood report mixed findings that differ by age, by the spatial skills assessed and by the tasks used (Newcombe, 2020). In a meta‐analysis, Lauer, Yhang, and Lourenco (2019) reported that small gender effects in mental rotation, that favour males, may be present from 6 years (Carr, Steiner, Kyser, & Biddlecomb, 2008). There is evidence that these gender differences may also be sensitive to development. For example, Neuburger, Jansen, Heil, and Quaiser‐Pohl (2011) found small gender effects in mental rotation favouring boys at 10, but not at 8 years. Other intrinsic‐dynamic tasks such as mental folding (Harris et al., 2013), mental transformation (Frick, 2019), and pattern construction (Gilligan, Flouri, & Farran, 2017) do not show a male advantage in middle childhood. Indeed, Gilligan et al. (2017) reported a small female advantage in pattern construction at 7 years. Prior research indicates that performance on the Children’s Embedded Figures Task (Witkin et al., 1971) shows either no gender differences in children aged 5–10 (Morris, Farran, & Dumontheil, 2019), or a female advantage in children aged 8‐to‐9 years (Kaplan & Weisberg, 1987). For extrinsic tasks, no gender differences have been reported in spatial perspective taking (Frick, 2019; Frick et al., 2014) or spatial scaling (Frick, 2019) in primary school‐aged children. Taken together, although some prior research highlights gender effects in spatial performance that may increase through development, the size of these effects is typically small in primary school‐aged children (Nazareth, Huang, Voyer, & Newcombe, 2019; Newcombe, 2020). Furthermore, an increasing number of studies do not report gender‐based effects. However, given that spatial task performance has sometimes been shown to differ by gender in middle childhood, it is important to account for this effect to provide an accurate analysis of age‐based differences.

### Current study

In the current study, we determine whether there are significant age‐based differences in intrinsic versus extrinsic, and static versus dynamic spatial skills, respectively. The findings from this study will provide a novel source of evidence to assess the spatial dimensions of the Uttal et al. (2013) model. Individual tasks were included to assess each of Uttal et al.'s (2013) proposed spatial dimensions (Figure 1). Our analysis contrasts with existing studies to date which test the model using factor analysis. We have two main hypotheses. First, we predict significant age‐based differences in intrinsic versus extrinsic spatial skills, with extrinsic skills continuing to develop into later childhood (significant age‐based differences between older age groups), even after 2D intrinsic skills have been acquired^2^ (no significant age‐based differences after 8 years). Second, we predict significant age‐based differences in static (significant age‐based differences between younger age groups only) and dynamic (significant age‐based differences between younger age groups as well as significant differences between older age groups) spatial skills. Finding a significant difference in the developmental trajectories of intrinsic versus extrinsic, and static versus dynamic skills, would support both dimensions of the Uttal et al. (2013) model.

## Materials and methods

### Participants

Participants were 185 children from a large, culturally diverse primary school in the UK. The eligibility for free school meals was 19%, slightly above the national average of 15% (Department for Education, 2019). Due to technical errors, 6 participants did not have a full set of scores available for analysis. Five of these participants were missing data for one task only, and to maximize statistical power, their missing scores were substituted using mean replacement, that is, replacing their missing score with the mean value on that task for their age group (missing data: two mental folding scores, two perspective taking scores, one CEFT score). The proportion of data replaced by mean scores was 0.005%. The sixth participant was missing data for several variables and was excluded. The final sample therefore consisted of 184 participants across six age groups (see Table 1). A power analysis, conducted in GPower, indicated that a total sample size of 140 was needed to detect a medium to large effect (0.3) for the main developmental analyses (one‐way ANOVA’s with age as a between participant variable; power = 0.8, α = .05; Table 1).

**Table 1:** Demographic information of participants across age groups

Age group	*N*	% Male	Age years (mean ± *SD*)
6 years	30	53.33	6.00 ± 0.34
7 years	31	41.94	6.99 ± 0.29
8 years	32	56.25	8.03 ± 0.28
9 years	31	45.16	8.97 ± 0.32
10 years	31	51.61	9.95 ± 0.33
11 years	29	58.62	11.00 ± 0.30

### Procedure

This study was part of a larger investigation of the role of spatial thinking for mathematics and science. More information can be found in Gilligan et al. (2019) and Hodgkiss et al. (2018). Each participant completed five testing sessions in a set order. There was approximately 2 days between testing sessions. The Mental Rotation Task and the Mental Folding Task were completed in a group testing session (8 participants per group), lasting approximately 35 min. The order of these tasks was counterbalanced. Each group testing session was supervised by at least two researchers. The CEFT, the Perspective Taking Task, and the Scaling Task were completed in an individual testing session, lasting approximately 45 min. The order of these tasks was counterbalanced. In the remaining three sessions, participants completed mathematics and science tasks, not described here.

### Measures

#### Children’s Embedded Figures Task—CEFT

The Children’s Embedded Figures Task (CEFT; Witkin et al., 1971) is a paper‐based task that uses physical shapes. The task requires participants to locate a target shape within a more complex figure. The task was administered as per the administration guidelines (Witkin et al., 1971). The task contained a maximum of two blocks, presented in a fixed order. Participants were first shown the target shape, a ‘tent’ shape for block A, and a ‘house’ shape for block B. Before the practice and experimental trials, participants were familiarized to the shape through four discrimination trials, where they were required to identify the target shape from a selection of other shapes. Participants repeated the discrimination trials until two items were answered correctly in succession. After this, participants completed either two (block A) or one (block B) practice trials, where they located the target shape hidden within a more complex image. Participants outlined the shape with their finger to indicate their answer. Participants were required to successfully locate the target shape in each practice item, before progressing to the experimental trials. Following the practice trials, participants completed 11 experimental items in block A and 14 experimental items in block B. For experimental items, they again were required to locate the target shape hidden with a larger more complex image. Performance was measured as percentage accuracy. Cronbach’s alpha for this task is high: .86 in children aged 6–11 years (Amador‐Campos & Kirchner‐Nebot, 1997).

#### Mental Rotation Task

**Figure 2:**
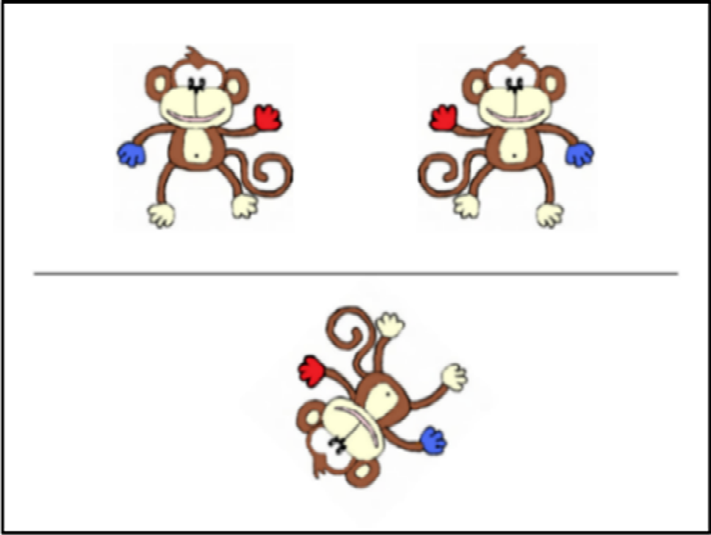
135° anticlockwise mental rotation trial.

**Figure 3:**
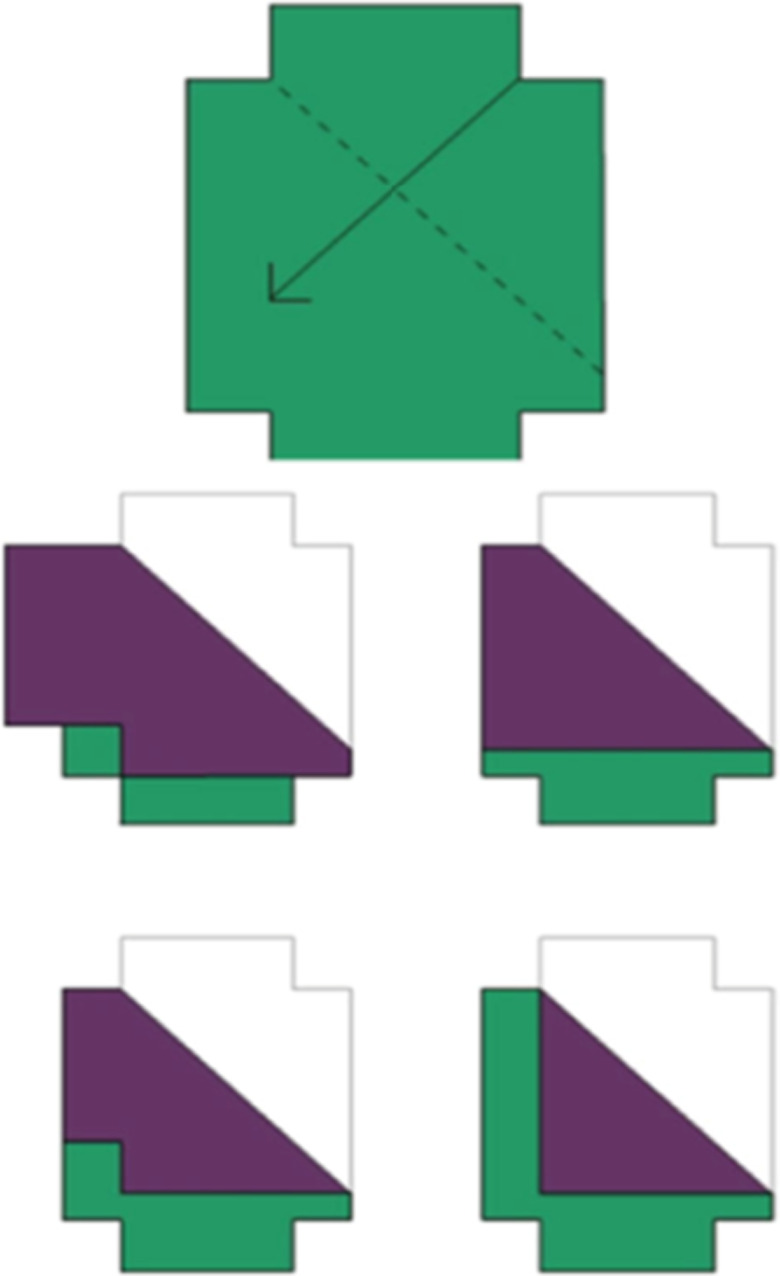
Sample trial from the Mental Folding Task.

This task was modified from Broadbent et al. (2014). Participants were required to identify which of two monkeys above a horizontal line displayed on screen, matched the target monkey below the horizontal line (see Figure 2). One monkey above the line was rotated by a fixed degree, relative to the target monkey. One monkey above the line was a mirror image of the target monkey. Participants indicated their answer using labelled keys on the computer keyboard. Participants completed four practice trials at 0°, that is, where the monkey below the horizontal line was not rotated. All participants passed the practice trials, that is, achieved 50% or higher on their first attempt. Participants next completed 36 experimental trials, 8 × 45° trials, 8 × 90° trials, 8 × 135° trials, and 8 × 180° trials and 4 trials at 0°. Equal numbers of clockwise and anticlockwise rotations were included. Performance was measured as percentage accuracy. Cronbach’s alpha reliability score for this task was high, α = .88 (Field, 2013; Figure 3).

#### Mental Folding Task

This task was taken from Harris et al. (2013). Participants were required to imagine folds made to a shape presented onscreen, without the physical representation of the fold itself (a physical piece of paper). Participants were shown a green shape on a computer screen (see Figure 2). The shape included a dotted line, which represented the folding line, and an arrow, which indicated the direction and distance of the required fold. Below this shape were four possible response options. Only one of these four response options (the target item) showed the outcome of the fold correctly. Participants completed two practice items, in which they were given a physical card to check their answer. If a child indicated an incorrect option, they were given one further attempt to answer the practice items. Most participants answered their practice items correctly on the first attempt and all participants answered correctly by the second attempt. Following the practice items, participants completed 14 experimental items. Performance was measured as percentage accuracy. Cronbach’s alpha reliability score for this task was medium/high, α = .74 (Field, 2013).

#### Spatial scaling

**Figure 4:**
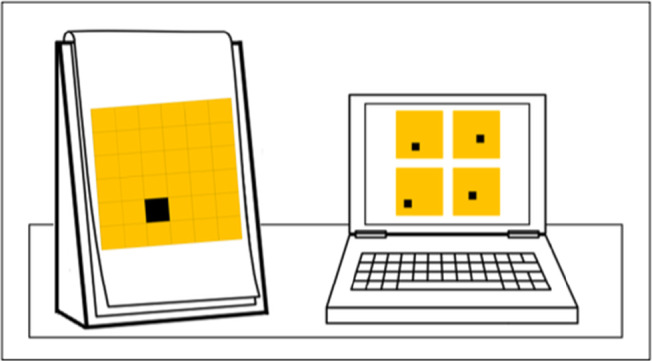
Spatial scaling trial at a scaling factor of 1:4.

In this task, children were required to locate the corresponding locations of hidden treasure (a black box) on two maps, when one was varied in size relative to the other (Gilligan et al., 2018). Participants were shown four treasure maps on a touch screen computer. To the left of the computer, children were presented with a printed treasure map (Figure 4). The child had to determine which of the four maps on the computer screen had the treasure positioned in the same place as the larger printed map. The three incorrect options were created uniformly for each trial. Trials differed by scaling factor. The printed maps were either unscaled with a ratio of 1:1 (7 cm × 7 cm), or, were scaled, to either a ratio of 1:2 (14 cm × 14 cm) or 1:4 (28 cm × 28 cm), relative to the maps on the computer (7 cm × 7 cm). The required level of visual acuity also differed across trials. At each scaling factor, the overall area of the maps, and by extension the scaling factor, did not change. However, half of the items were presented using a 6 × 6 square grid and therefore required gross level acuity, while the other half were presented using a 10 × 10 square grid and therefore required fine level acuity. Participants first completed two unscaled practice items. All participants passed the practice trials, that is, answered at least one of the practice items correctly on their first attempt and thus continued to the main trials. Participants completed 18 experimental trials including six items at each scaling factor. Performance was measured as percentage accuracy. Cronbach’s alpha reliability score for this task was medium/high, α = .74 (Field, 2013).

#### Perspective taking task for children

**Figure 5:**
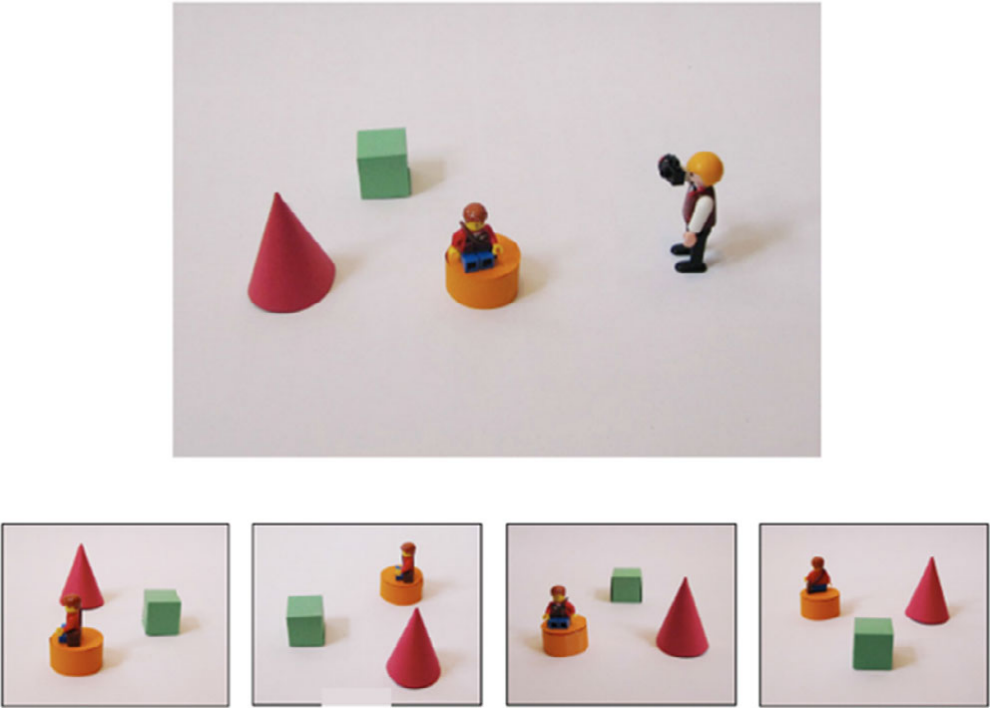
Perspective taking, 90°, three object trial.

In this task, participants visualised what photographs would look like when taken from cameras placed at different angles and positions relative to their viewpoint (Frick et al., 2014; Figure 5). Participants completed four practice questions with physical Playmobil characters (one holding a camera) in a specified arrangement on a table. For each practice trial, the participant was shown a photograph and was asked which of the two characters could have taken the photograph. Participants were able to check their answers by moving around the table to be positioned at the photographer’s perspective. Feedback was given on each practice item. If a participant made an error on any of the practice items, they were allowed a maximum of one additional attempt. Few participants made errors on the first attempt and all participants passed on their second, if one was needed. Participants completed 18 experimental trials. The angular difference between the photographer’s and the participant’s perspective varied across experimental trials (0°, 90° or 180°). The number of objects in the layout (1, 2, or 3) also varied across experimental trials. Cronbach’s alpha reliability score for this task was high, α = .84 (Field, 2013).

### Analysis strategy

There were no significant floor or ceiling effects for any task, for any age group. We then created intrinsic, extrinsic, static, and dynamic composite variables by calculating the mean *z*‐score for the tasks which were categorized as assessing that spatial dimension: intrinsic (CEFT, mental rotation, and mental folding); extrinsic (spatial scaling and perspective taking); static (CEFT and spatial scaling); and dynamic (mental rotation, mental folding and perspective taking; see Figure 1).

All variables were broadly normal based on visual inspection of Q–Q plots and boxplots. Gender effects were investigated through Bonferroni‐corrected *t*‐tests for each spatial dimension (0.05/4 = .013). We ran two mixed ANOVAs to investigate age‐based differences in performance across spatial dimensions. Age group was the between‐group factor and the intrinsic and extrinsic composite variables (or static and dynamic composite variables respectively) were the within‐participant variables. An interaction between age and spatial dimension in either model would indicate that the trajectories of the spatial dimensions differed across age groups and would therefore support either or both dimensions of the Uttal et al. (2013) model.

Significant interactions were first explored by comparing the effect sizes of Tukey post‐hoc tests. Two follow‐up ANOVAs were also completed to statistically compare relatively younger and older children within two broader age categories. These ANOVAs only included children aged 6, 8, 9 and 11. The children were divided into two broad age categories: *Younger: 6‐ and 8‐year‐olds; Older: 9‐ and 11‐year‐olds*. The children were further categorized within these broad categories as being relatively younger or relatively older (e.g., within the broad category of younger, 6‐year‐olds were coded as relatively younger and 8‐year‐olds as relatively older). A significant interaction between broad and relative age grouping would indicate that age‐based differences were greater within one of the broader age categories than the other.

## Results

### Analysis of gender effects

After applying a Bonferroni correction (0.05/4 = .013), there were no significant gender differences in spatial performance for any of the spatial dimensions (*p* > .013; *d* < 0.360 for all). In addition, no interactions between gender and age were found when gender was included within the main ANOVAs, reported below (*p* > .05; $\eta_{p}^{2}$ < .005 for all). Gender was therefore not included as a factor in subsequent analyses.

### Age‐based differences in intrinsic versus extrinsic spatial dimension

**Figure 6:**
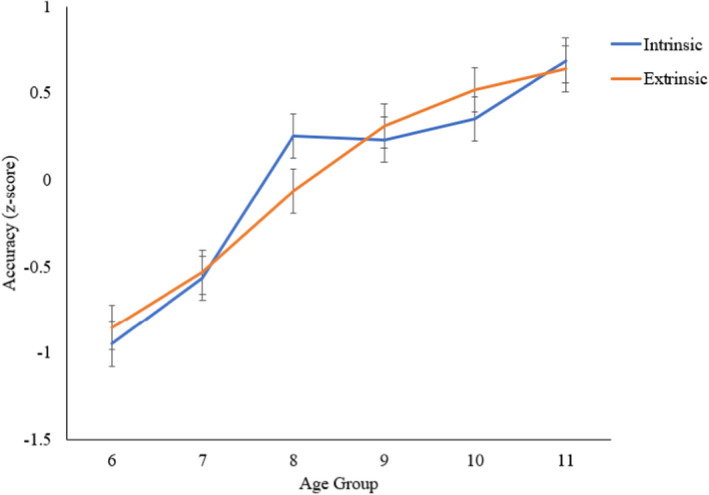
Intrinsic and extrinsic composite score accuracy by age group.

Descriptive statistics for each spatial task across each age group, including both raw scores and *z*‐scores, can be found in the Supporting Information. For the ANOVA comparing the intrinsic versus extrinsic spatial dimension by age group, there was a main effect of age group, *F*(5, 178) = 32.758, *p *< .001, $\eta_{p}^{2}$ = .479, and a significant interaction between age group and spatial dimension, *F*(5, 178) = 2.577, *p *= .028, $\eta_{p}^{2}$ = .068 (Figure 6). Tukey post‐hoc tests revealed subtle differences in the development of spatial skills across the intrinsic and extrinsic spatial dimension (see Table 2 for full results). For the youngest children, performance increased significantly between 6 and 8 years for both intrinsic (*p *< .001) and extrinsic skills (*p *< .001), although the effect was larger for the intrinsic (*d* = 2.047), than the extrinsic dimension (*d* = 1.105). Between 7 and 8 years, there was a significant improvement in performance for intrinsic (*p *< .001*, d* = 1.393) but not extrinsic skills (*p *= .103, *d *= 0.655). For older age groups, there was a significant increase in performance between 8 and 11 years for both intrinsic (*p *= .048; *d *= 0.743) and extrinsic skills (*p =* .002; *d *= 0.988). Between 9 and 11 years, there was a significant difference in performance for intrinsic (*p =* .035, *d *= 0.777) but not extrinsic skills (*p *= .49, *d *= 0.457*)*. In contrast, there was a significant increase between 8 and 10 years for extrinsic skills (*p =* .017, *d *= 0.820) but not intrinsic skills (*p *= .99, *d *= 0.165). Overall, the interaction between age and spatial dimension was driven by a steep rate of early development for intrinsic skills and a slower and later development of extrinsic skills.

**Table 2:** Summary of Tukey post‐hoc tests by age group

Comparison		Intrinsic			Extrinsic		
Age groups		Mean difference	Tukey, *p*	Tukey, *d*	Mean difference	Tukey, *p*	Tukey, *d*
6	7	−0.38	.115	−0.654	−0.32	.496	−0.450
	8	−1.20	<.001	−2.047	−0.79	<.001	−1.105
	9	−1.18	<.001	−2.012	−1.17	<.001	−1.635
	10	−1.30	<.001	−2.212	−1.37	<.001	−1.925
	11	−1.64	<.001	−2.789	−1.49	<.001	−2.093
7	8	−0.82	<.001	−1.393	−0.47	.103	−0.655
	9	−0.80	<.001	−1.358	−0.85	<.001	−1.185
	10	−0.91	<.001	−1.558	−1.05	<.001	−1.475
	11	−1.25	<.001	−2.136	−1.17	<.001	−1.643
8	9	0.02	1.000	0.035	−0.38	.290	−0.530
	10	−0.10	.987	−0.165	−0.59	.017	−0.820
	11	−0.44	.048	−0.743	−0.70	.002	−0.988
9	10	−0.12	.970	−0.200	−0.21	.863	−0.290
	11	−0.46	.035	−0.777	−0.33	.487	−0.457
10	11	−0.34	.226	−0.578	−0.119	.987	−0.167

The additional ANOVAs exploring aged‐based differences for younger versus older children revealed for intrinsic skills a significant interaction between broad and relative age groupings, *F*(1, 118) = 13.8, *p *< .001, $\eta_{p}^{2}$ = .105. The interaction was driven by the difference between the 6‐ and 8‐year‐olds (−1.20) being significantly greater than the 9‐ and 11‐year‐olds (−0.46). For extrinsic skills, there was no significant interaction between broad and relative age groupings, *F*(1, 118) = 3.07, *p* = .082, $\eta_{p}^{2}$ = .025.

### Age‐based differences in static versus dynamic spatial dimension

**Figure 7:**
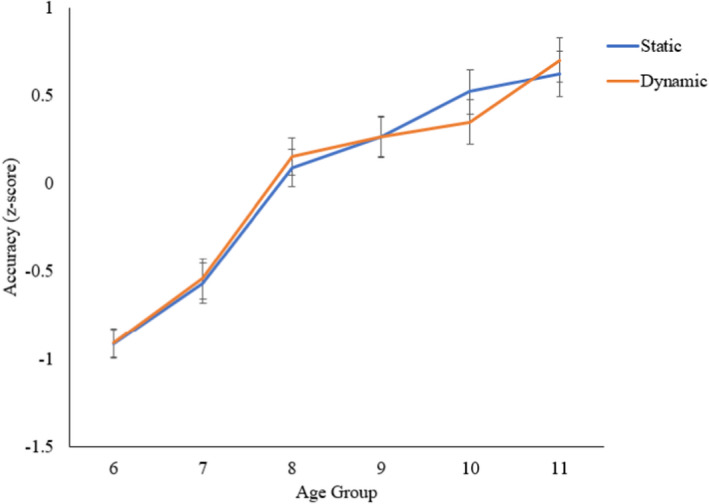
Static and dynamic composite score accuracy by age group.

For the ANOVA of static versus dynamic skills by age group, there was a main effect of age group, *F*(5, 178) = 34.190, *p *< .001, $\eta_{p}^{2}$ = .490; the interaction between age group and spatial dimension was not statistically significant, *F *< 1 (Figure 7).

## Discussion

This study is the first to use developmental trajectories as a source of evidence to assess the spatial dimensions of the Uttal et al. (2013) model. Subtle differences in the development of intrinsic versus extrinsic spatial skills were found. This was not the case for the static versus dynamic dimension. The findings in the current study complement prior psychometric data, for example, Mix et al. (2018) which reported a distinction between intrinsic versus extrinsic spatial skills, but not between static versus dynamic spatial skills.

Age‐based differences were found for extrinsic compared to intrinsic spatial skills. Analysis of effect sizes suggested that the differences in trajectories were driven by a steep rate of early development for intrinsic skills and a slower and later development for extrinsic skills.

Performance increased more between 6 and 8, and 7 and 8 years, for intrinsic spatial skills, than for extrinsic spatial skills. In contrast, extrinsic spatial performance differed more between 8 and 10 years, compared with intrinsic spatial performance. There was no evidence that the trajectories of static versus dynamic skills differed significantly. This contrasted with our hypothesis that static skills (perceiving and encoding static images) may be a prerequisite for dynamic skills. Therefore, while static skills (e.g., disembedding) may show earlier development than dynamic skills (e.g., mental rotation) in early childhood, between 3 and 6 years (Busch et al., 1993; Witkin et al., 1971), there is no evidence from our study that this developmental dissociation continues into middle childhood. Alternatively, the static and dynamic tasks that were included in our study may not have been sensitive enough to highlight subtle developmental differences beyond 6 years.

The differing developmental patterns between intrinsic and extrinsic spatial skills lends support to the intrinsic versus extrinsic spatial dimension in Uttal et al.'s (2013) model of spatial thinking. The results reported here show that performance on tasks measuring the intrinsic versus extrinsic spatial dimension differ developmentally for intrinsic and extrinsic skills, thus suggesting that they are distinct constructs. The results align with the aforementioned CFA study by Mix et al. (2018) that found stronger evidence for the intrinsic versus extrinsic, compared to the static versus dynamic distinction of spatial thinking. These findings are also consistent with previous research that demonstrated that the developmental trajectories of mental rotation (intrinsic skill) and perspective taking (extrinsic skill) differed such that mental rotation developed earlier (from age 7 years) than perspective taking (from age 8 years; Crescentini et al., 2014). Here, we extend these findings and show earlier development of intrinsic compared to extrinsic skills using more comprehensive measures of the intrinsic versus extrinsic dimension.

Although significant age‐based differences are reported in this study, the findings also highlight substantial individual differences in spatial performance (reflected by the range of z‐scores, standard deviations, and standard errors) for both of Uttal et al.'s (2013) spatial dimensions. The roles of both development and individual differences must be considered in any discussion of spatial thinking. For example, one demographic factor that was explored in this study was gender. While some previous studies have outlined a male advantage in spatial task performance in childhood (Carr et al., 2008; Casey et al., 2008; De Lisi & Wolford, 2002; Lauer et al., 2019; Neuburger et al., 2011; Wiedenbauer & Jansen‐Osmann, 2008), in this study no significant gender differences were found and all effect sizes reported for gender were small (*d* < 0.360). This finding is consistent with other recent studies that do not report a male advantage for spatial outcomes (Frick, 2019; Frick et al., 2014; Gilligan et al., 2017; Harris et al., 2013).

This study is not withstanding limitations. Owing to the cross‐sectional design, it was not possible to compare individuals’ performance across time. These findings could be strengthened by replication using a longitudinal design. The findings of this study provide a comparison of spatial performance between spatial dimensions, in this age range, for the first time. From a practical perspective, the results highlight developmental ages when spatial tasks may be particularly challenging and when scaffolding or spatial training, in specific skills, may be particularly beneficial.

To conclude, this is the first study to use developmental trajectories as a source of evidence to assess the Uttal et al. (2013) model. We showed that there were age‐based differences in intrinsic and extrinsic spatial performance, such that intrinsic skills demonstrated particularly rapid early development in middle childhood (6–8 years) compared to extrinsic skills. In contrast, there were larger differences in extrinsic spatial performance between 8 and 10 years. There were no age‐based differences in static versus dynamic spatial performance. The findings therefore lend support to the intrinsic versus extrinsic dimension of the Uttal et al. (2013) model only. By exploring spatial performance in older children, the results demonstrate for the first time that some spatial subdomains continue to develop at least until 11 years.

## Conflicts of interest

All authors declare no conflict of interest.

## Author contributions

Alex Hodgkiss (Conceptualization; Formal analysis; Investigation; Methodology; Project administration; Writing – original draft; Writing – review & editing). Katie A. Gilligan‐Lee (Conceptualization; Formal analysis; Investigation; Methodology; Project administration; Writing – original draft; Writing – review & editing). Michael S. C. Thomas (Conceptualization; Supervision; Writing – review & editing). Andrew K Tolmie (Conceptualization; Supervision; Writing – review & editing). Emily K Farran (Conceptualization; Methodology; Supervision; Writing – review & editing).

## Supporting information


**Table S1**. Descriptive statistics for each spatial task by age group (raw scores).
**Table S2**. Descriptive statistics for each spatial skill by age group (*z*‐scores).

## Data Availability

Data available on request from the authors.

## Footnotes

^1^
The oldest age group of children included in this study was 8 years.

^2^
3D rigid intrinsic‐dynamic skills, which were not tested in this study, continue to develop into late childhood (Johnson & Meade, 1987); this prediction therefore applies only to 2D skills.
