# Supplementary material for: The developmental trajectories of spatial skills in middle childhood
Source: Br J Dev Psychol. 2021 May 18;39(4):566–83. doi: 10.1111/bjdp.12380 (PMC8519147; doi:10.1111/bjdp.12380)
Supplement: Supplementary file 1 — Table S1. Descriptive statistics for each spatial task by age group (raw scores). Table S2. Descriptive statistics for each spatial skill by age group (z‐scores). [file BJDP-39-566-s001.docx]

**Supplementary Material**

Table S1: Descriptive statistics for each spatial task by age group (raw scores)

|  | **Min** | **Max** | **6 years** | **7 years** | **8 years** | **9 years** | **10 years** | **11 years** |
| --- | --- | --- | --- | --- | --- | --- | --- | --- |
| Embedded figures (I/S) | 1 | 23 | 7.66 (3.12) | 8.97 (3.34) | 12.72 (4.11) | 12.68 (3.99) | 14.13 (4.49) | 15.17 (4.13) |
| Mental folding (NR- I/D) | 1 | 14 | 5.21 (2.07) | 6.06 (2.93) | 9.28 (2.19) | 8.61 (3.28) | 9.29 (2.51) | 10.32 (2.63) |
| Mental rotation (R-I/D) | 2 | 40 | 23.53 (4.75) | 27.68 (6.94) | 32.09 (6.08) | 33.18 (4.64) | 32.00 (7.33) | 35.24 (3.99) |
| Spatial scaling (E-S) | 2 | 18 | 6.80 (2.51) | 8.32 (3.52) | 10.22 (3.54) | 11.58 (2.86) | 12.32 (2.66) | 12.24 (3.55) |
| Perspective taking (E-D) | 3 | 18 | 7.86 (2.49) | 8.77 (2.94) | 10.44 (3.20) | 11.97 (3.77) | 12.81 (3.66) | 13.83 (3.80) |

*Note:* Raw scores (Mean [Standard Deviation]) are reported.

I/S: Intrinsic/Static NR-I/D: Non-rigid intrinsic/dynamic R-I/D: Rigid-intrinsic/dynamic E-D: extrinsic/static E-D: extrinsic/dynamic

Table S2: Descriptive statistics for each spatial skill by age group (z-scores)

|  | **Min** | **Max** | **6 years** | **7 years** | **8 years** | **9 years** | **10 years** | **11 years** |
| --- | --- | --- | --- | --- | --- | --- | --- | --- |
| Intrinsic | -1.67 | 1.65 | -0.95 (0.43) | -0.57 (0.66) | 0.25 (0.52) | 0.23 (0.63) | 0.35 (0.64) | 0.69 (0.61) |
| Extrinsic | -1.59 | 1.82 | -0.85 (0.48) | -0.53 (0.68) | -0.07 (0.73) | 0.31(0.75) | 0.52 (0.69) | 0.64 (0.89) |
| Static | -1.79 | 2.12 | -0.91(0.48) | -0.57 (0.73) | 0.09 (0.71) | 0.26 (0.69) | 0.52 (0.55) | 0.62 (0.84) |
| Dynamic | -1.73 | 1.67 | -0.91 (0.45) | -0.54 (0.64) | 0.15 (0.60) | 0.26 (0.64) | 0.35 (0.71) | 0.70 (0.69) |

*Note:* Z scores (Mean [Standard Deviation]) are reported.
